# Spinal Muscular Atrophy With Severe Hyperlordosis: A Case Report

**DOI:** 10.7759/cureus.53898

**Published:** 2024-02-09

**Authors:** Prachi Sharma, Sham Lohiya, Keta Vagha, Jayant D Vagha, Himanshu Raj, Roshan Prasad

**Affiliations:** 1 Pediatrics, Jawaharlal Nehru Medical College, Datta Meghe Institute of Higher Education and Research, Wardha, IND; 2 Pediatrics and Neonatology, Jawaharlal Nehru Medical College, Datta Meghe Institute of Higher Education and Research, Wardha, IND

**Keywords:** nusinersen, smn gene, kugelberg-welander disease, sma plus, hyperlordosis, spinal muscular atrophy

## Abstract

Spinal muscular atrophy (SMA) indicates a set of inherited autosomal recessive genetic disorders, where, specifically, the anterior horn cell motor neurons in the brain and spinal cord are affected, leading to a severe form of hypotonia and muscle weakness. The incidence is exceptionally rare, commonly manifesting as slowly progressive muscular weakness and atrophy of lower limbs. As per our existing knowledge, this is the first case of SMA associated with hyperlordosis in a patient. Hyperlordosis is a deformity in spinal curvature characterized by an excessive forward spinal curve in the region of the lower back, forming the characteristic C-shape curvature in the lumbar region, just above the buttocks.

Parents brought an 11-year-old male child with complaints of inability to get up from a sitting position along with difficulty in walking for the past six months. Upon physical examination, deep tendon reflexes were absent; there was severe hyperlordosis, proximal limb weakness, and notable hypotonia. In our study, we aim to understand the clinical presentation, impact, and association of hyperlordosis in a child diagnosed with SMA. This case report describes the complaints and successful diagnosis of a patient of survivor motor neuron (SMN) gene-related SMA along with severe hyperlordosis backed by evidences of electrophysiology and neuropathology. However, a complete cure and normal lifestyle are not possible due to the lack of affordable and easily accessible therapies.

## Introduction

Spinal muscular atrophy (SMA) is a neuromuscular disorder specifically known as neuronopathy. It affects the motor neurons of anterior horn cells of the central nervous system. Motor neurons are responsible for skeletal muscle activity of the body [1]. SMA has an autosomal recessive (AR) mode of inheritance, thus affecting both genders equally, with an incidence of one in 10,000 worldwide [1-3]. It is a severe genetic disorder affecting children in the early years of life and a key cause of death in infancy. SMA occurs because of a genetic defect in the survivor motor neuron (SMN) gene located on the long arm of chromosome 5 (5q). There are two types of SMN genes: 1 and 2. The SMN1 gene codes for the functional protein, while the SMN2 gene codes for partly functional or dysfunctional proteins (non-functional proteins) due to defective exons [1,2].

The normal function of the SMN gene is to inhibit the programmed apoptosis of motor neurons, thereby maintaining their normal function and prolonging the life of motor neurons. However, due to a defective SMN gene, the rate of apoptosis of motor neurons increases, which causes no impulse to reach the muscles and causes flaccidity or decreased muscle tone as the primary manifestation, thus resulting in loss of normal neuromuscular functions. Individuals identified with SMA have lower-than-normal amounts of the SMN protein, which causes the death of motor neurons in both the central and peripheral nervous systems, ultimately causing atrophy of the skeletal muscles, which is more severe in the trunk (chest), proximal lower limb, and upper limb muscles than in the distal muscles of hands and feet [1,3].

SMA does not affect the sensory nerves and intellectual status of the patient. Rather, it has been observed that many patients with SMA are highly intelligent. Usually, spine deformities like scoliosis are seen in patients with SMA [4]. However, this case report shows the rare and unique presentation of SMA in a child with severe hyperlordosis, which will highlight its cause, its myriad manifestations, and the need for an affordable and accessible approach in terms of its management, both investigations and treatment.

## Case presentation

An 11-year-old male child of the Hindu religion, born out of a non-consanguineous marriage, is brought by the father with complaints of difficulty in getting up from a sitting position and difficulty in walking for six months. As narrated by the father, the child was apparently alright till one year of age, when he developed weakness in the lower limbs, for which he was taken to a private hospital where some oral medications were given. When the child was around one year old, the father also noticed that the child could not stand with support. At 18 months, the father noticed that the child was unable to stand without support. The child attained walking at a much later age than the children of his age. Antenatal, natal, and postnatal history were non-eventful.

**Figure 1:**
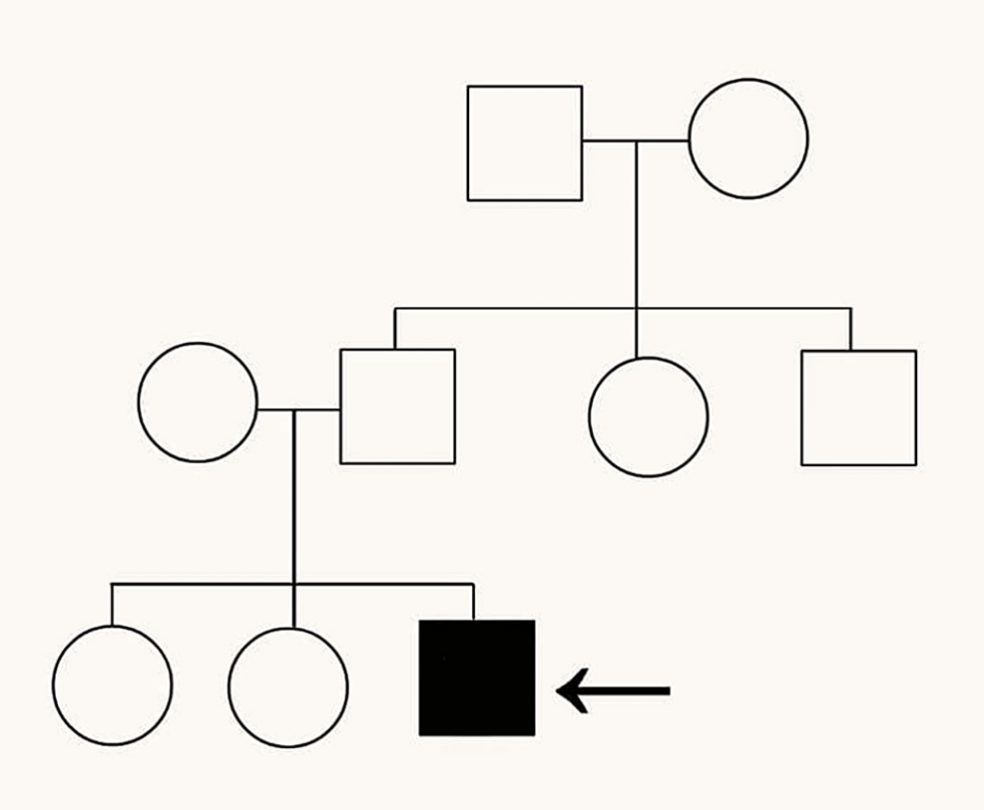
Family pedigree of the patient. The arrow shows the index case, which here is the patient.

**Figure 2:**
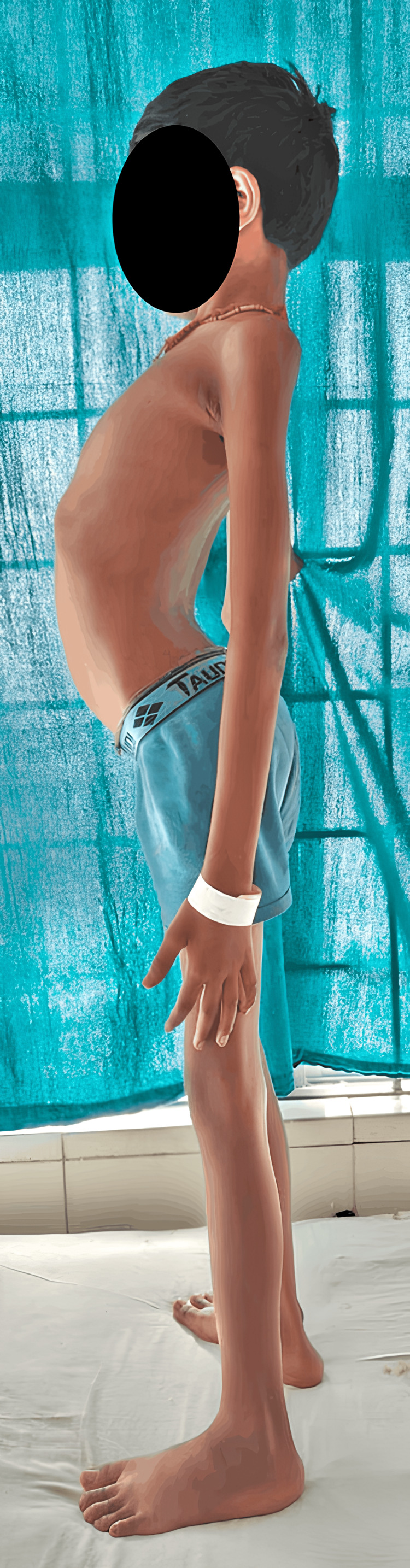
Increased curvature of the spine towards the front showing hyperlordosis.

**Figure 3:**
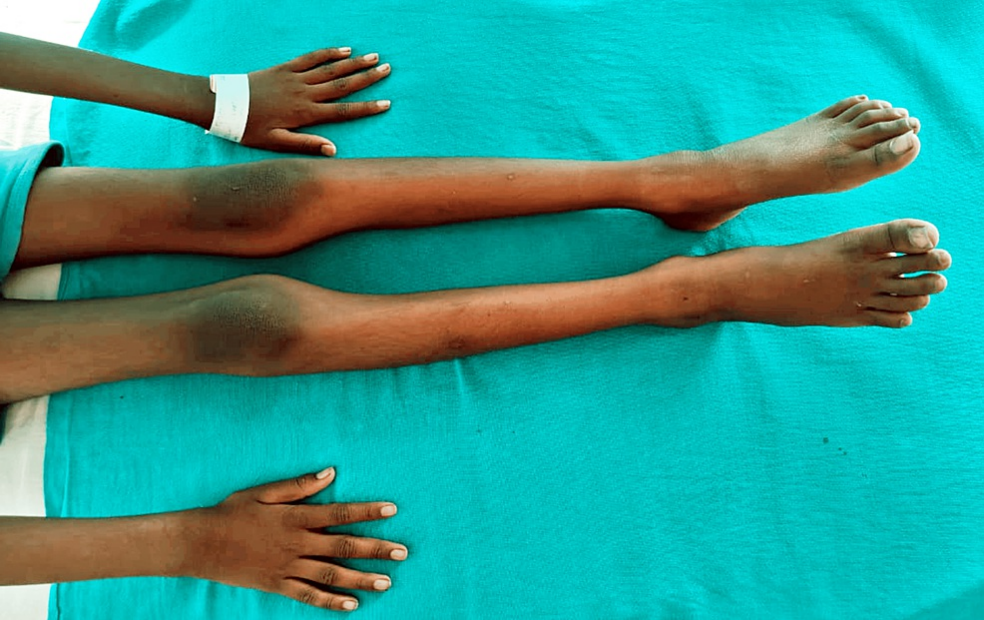
Diffuse muscle wasting involving both upper and lower limbs.

As the child has grown, he has further deformities of the knee and chest, as per the father. At present, there is no history of (h/o) pain, fever, recurrent infections, or frequent hospital admissions. Currently, the child is able to carry out daily activities like wearing clothes and combing hair with little help. He faces difficulty in walking and getting up from a sitting position, which has increased over the past six months. After receiving the child's complaints, the parents approached the hospital for further treatment. He is referred to the rehabilitation and neurology department. The child is intellectually normal. On examination, the child is conscious, cooperative, and well-oriented to time, place, and person. There are no complaints regarding sensory or cognitive impairment, ataxia, or bladder-bowel involvement. Family history is non-contributory, and a family tree depicting the pedigree has been provided in Figure 1. Examination shows pectus excavatum and an increase in the curvature towards the front of the body in the lumbar spine region, as seen in Figure 2. He also has genu recurvatum, diffuse muscle wasting, and lower motor neuron (LMN) type of upper and lower limb weakness, as seen in Figure 3.

Deep tendon reflexes are attenuated globally which according to the grading system was found to be of grade 1, and the Gowers sign is positive. His temperature is 98° Fahrenheit, heart rate is 90 beats/min, respiratory rate is 22/min, oxygen saturation is 97%, and random blood sugar is 80 mg/dl. The anthropometric measurements reported a height of 76 cm, weight of 7 kg, head circumference of 50 cm, and body mass index (BMI) of 11.77. Routine investigations are done, the results of which are mentioned in a tabular form in Table 1 [5].

**Table 1:** Lab reports of the patient and normal values. RBC: red blood cell; WBC: white blood cell; ALP: alkaline phosphatase; AST: aspartate aminotransferase; ALT: alanine transaminase

Investigation	Results	Normal value
Total RBC count (millions/mm³)	5.41	4-5.5
Hemoglobin (g/dl)	12.2	12.5-15.5
Hematocrit (%)	37.2	36-47
Mean corpuscular volume (fL)	68.7	78-95
Mean corpuscular hemoglobin (pg/cell)	22.5	26-32
Mean corpuscular hemoglobin concentration (g/dL)	32.8	30-36
Red cell distribution width	16.2	<14.5
Total WBC count (/mm³)	14100	4000-13500
Total platelet count (lakh/µL)	3.58	1.5-4.5
Calcium (mg/dL)	10.6	8.8–10.8
Sodium (mEq/L)	137	130-147
Potassium (mEq/L)	4.3	3.5-5.1
Phosphorus (mg/dL)	7	3.2-5.8
Urea (mg/dL)	23	5-20
Creatinine (mg/dL)	0.3	0.5-1
Creatinine kinase (IU/L)	106	5-200
ALP (IU/L)	236	140-560
AST (IU/L)	31	10-40
ALT (IU/L)	26	5-55
Total bilirubin (mg/dL)	0.6	<1.5
Vitamin D (IU/L)	33.2	30-100
Total protein (g/dL)	7.5	6-8

As per the World Health Organization (WHO) classification, the weight for height was less than -3 standard deviation (SD), and the height for age was less than -3 SD. A syrup containing coenzyme Q10 and levocarnitine is started. Type III SMA is suspected, and molecular genetic testing is requested. A homozygous deletion in exons 7 and 8 of the SMN1 gene was identified. The patient is diagnosed with type III SMA, which is also known as the Kugelberg-Welander disease.

The patient is recommended for nusinersen as the definitive treatment, but due to financial restraints, it is denied by the patient's parents. Currently, the child is offered symptomatic treatment by providing a healthy balanced diet and treating or preventing complications of weakness, along with neurophysiotherapy and occupational physiotherapy. He has been advised to regular follow-up.

## Discussion

This report highlights a rare case of SMA combined with severe hyperlordosis. Based on the child's age during the onset of symptoms and the maximum motor function achieved, SMA is mainly of five clinical types (Table 2) [1,6].

**Table 2:** Types of SPA and its features. Image Credit: Author References: [1,6] SMA: spinal muscular atrophy

Types	Onset	Presentation
0	Prenatal	Fetal or intrauterine death.
I (classical)	0-6 months	Also referred to as infantile-onset SMA or Werdnig-Hoffmann illness. The children who have been affected experience a reduced range of motion and develop contractures due to the shortened length of muscles or tendons. The infant adopts a frog-like stance. It is possible to see reduced muscular tone, absent tendon reflexes, twitching, musculoskeletal abnormalities, and difficulties eating and swallowing. When left untreated, many afflicted kids pass away before turning three.
II	6-18 months	Also known as Dubowitz illness. Affected children need assistance to stand or walk, but they can sit without support. Scoliosis and respiratory issues are other possible conditions in these children. Although life expectancy is declining, the majority of people reach school age.
III	>18 months	Also known as Kugelberg-Welander disease. Although they can walk independently, children may find it challenging to run, get out of a chair, or ascend stairs. Respiratory infections, contractures, and spinal curvature are possible additional problems. Most people can live typical lives with therapy.
IV	>21 years	Without affecting longevity, children experience modest to severe weakening in their leg muscles along with other minor symptoms.

Our patient was diagnosed with type III SMA, also known as "walkers" or mild SMA. It manifests as gradually increasing weakness of the proximal region of limbs, which affects the lower limb muscles more than the upper limb. However, the patient is able to stand and walk with little support. A tiny subset of patients experienced hip subluxation, scoliosis, and an elevated risk of fractures in addition to restrictive lung illness [7-9].

Through thorough history taking, physical examination, and investigations like nerve conduction studies, creatinine kinase (CK), electromyelogram (EMG), muscle biopsy, and magnetic resonance imaging (MRI), the diagnosis of Kugelberg-Welander disease can be made. The definite diagnosis of SMA is done by molecular genetic testing [8]. Genetic testing of SMN1/2 is considered the first line of investigation, and there is no requirement for an invasive test like muscle biopsy.

Previously, patients with SMA were primarily offered supportive treatment with the early engagement of pediatric specialists. Recently developed novel therapies show significant promise in addressing historically poor morbidity and mortality rates [9-11]. Non-invasive ventilation, usually in the form of bilevel positive airway pressure (BiPAP), has improved both quality of life and life expectancy when restrictive lung conditions appear. However, this is only a tiny fraction of type III SMA patients [12]. People who need this kind of assistance frequently have a weak cough and are more likely to experience respiratory issues such as aspiration, mucus plugging, recurring infections, and hypoxemia. When it comes to evaluating cough function, removing mucus, and keeping an eye on forced vital capacity in children older than five, the cooperation of chest physiotherapists is essential [1,8].

When non-invasive ventilation proves insufficient, tracheostomy and permanent invasive ventilation must be approached within a multidisciplinary framework with the help of palliative care specialists [12,13]. Due to associated muscle weakness, patients may experience rapid fatigue and difficulties with swallowing, exacerbating the impact of muscle weakness. Gastrointestinal symptoms, including constipation, delayed gastric emptying, and reflux (rarely seen in type III SMA), necessitate the essential involvement of nutritionists to ensure optimal nutritional management [14].

Patients with type III SMA frequently experience orthopaedic issues, including scoliosis, hip subluxation, and fracture susceptibility. Physiotherapy is crucial in optimizing and preserving function, utilizing stretching exercises and passive joint movements to prevent joint contractures. To improve quality of life and mobility, consultation with orthotic professionals is essential when installing frames, orthotics, and wheelchairs. For scoliosis, ongoing orthopaedic surgical surveillance is necessary, and bracing and spinal fusion should be periodically considered as therapies [6,15].

Novel medicines with the potential to improve longevity and reduce morbidity have been made possible by advances in genetic therapy and a deeper understanding of the underlying pathophysiology. The first medication licensed to treat SMA in adults and children is called nusinersen. Nusinersen is an antisense oligonucleotide (ASO), which, through the inhibition of ISS-N1, an SMN2 exon 7 splicer, facilitates the synthesis of functional SMN2. This approach results in increased synthesis of functional SMN protein and improved motor function for type III patients. With the exception of a few documented occurrences of constipation and respiratory tract infections, no substantial serious adverse effects were observed as a result of using this medication. Four initial loading doses are supplied by intrathecal injection; the fourth loading dosage is given 30 days following the third loading dose, and the first three loading doses are given at intervals of 14 days. A maintenance dosage is then administered once every four months following that. When available, therapy with nusinersen is advised for the majority of patients despite its high cost ($125,000/dose), as it can prevent the condition from getting worse and prevent respiratory failure and death [16-18]. Another drug, risdiplam, which modulates the splicing of SMN2 is also of importance in the treatment. It is cheaper and given orally which promises compliance in the patient population. Risdiplam was approved in 2020 to be used in patients suffering from SMA. Side effects such as constipation, vomitting, respiratory tract infections, and rhinitis were noted in approximately 10% of the patients; however, the long-term results are still limited. In spite of these novel drugs, an insufficient medical need in the patient population is observed. Importantly, the past decade has seen growing reports of recovery in the natural course of illness in all types of SMA cases [18,19]. Survival rates in type I SMA, the most severe form, have increased due to a proactive approach incorporating supportive ventilation and non-parenteral feedings. These favourable outcomes are mostly a result of recent advancements in treatment and support to the patient that may not be fully represented in the existing studies.

## Conclusions

In our case report, an 11-year-old male child presented with complaints of difficulty in getting up from a sitting position and difficulty in walking and had an increase in the curvature towards the front of the body in the lumbar spine region, pointing towards diagnosing SMA associated with hyperlordosis. There aren't many documented instances of these kinds of presentations, which makes this one special. Our goal in presenting this case study was to demonstrate that hyperlordosis is a possible symptom of SMA. Treating individuals with SMA efficiently requires an understanding of the underlying pathophysiology, subtypes, and new medications. The significance of the specialised departments in diagnosing and treating individuals with SMA is highlighted in this case report, which also discusses the examination and treatment of the disorder.
